# Supplementary material for: Phenolic-Rich Indian Almond (Terminalia catappa Linn) Leaf Extract Ameliorates Lipid Metabolism and Inflammation in High-Fat Diet (HFD)-Induced Obese Mice
Source: Metabolites. 2025 Sep 8;15(9):594. doi: 10.3390/metabo15090594 (PMC12471564; doi:10.3390/metabo15090594)
Supplement: Supplementary file 1 [file metabolites-15-00594-s001.zip › metabolites-3828470-supplementary.pdf]

Phenolic-rich Indian almond (*Terminalia catappa* Linn) leaf extract ameliorates lipid metabolism and inflammation in high-fat diet (HFD)-induced obese mice

Opeyemi. O. Deji-Oloruntoba 1†, Ji Eun Kim2†, Hee Jin Song2, Ayun Seol2, Dae Youn Hwang2 and Miran Jang3*

1 Biohealth Convergence Unit, Food, and Drug Biotechnology, Inje University, Korea; oodejiolorunto-ba@gmail.com
2 Department of Biomaterials Science (BK21 FOUR Program), Life and Industry Convergence Research Institute, College of Natural Resources and Life Science, Pusan National University, Miryang 50463, Republic of Korea
3 Department of Food and Drug Biotechnology, Inje University, Gimhae, 50834, Korea; mrjang@inje.ac.kr
* Correspondence: mrjang@inje.ac.kr; Tel.: +82-10-9984-4359
† Equal contribution

Table S1. UPLC-QTOF-MS analysis condition of phenolic compounds in TCE

**LC Condition (Waters® ACQUITYTM UPLC).**	
Column	CORTECSTM UPLC® C181.6μm(2.1 × 100mm) Temperature: 35 ◦C
Mobile phase	(A) 0.1% formic acid in water<br>(B) 0.1% formic acid in Methanol
Gradient	5% solvent B for 1 min, 5–30% solvent B for the next 4 min, 30–32% solvent B for 2 min, 32–50% solvent B for 5 min, and a linear step of 50–5% solvent B for 3 min for column re-equilibration.
Flow rate	0.25 mL/min
**MS Condition (SYNAPT™ G2)**	
Ionization Mode	ESI-
Temperature	Source: 120 ◦C, and desolvation: 350 ◦C
Voltage	Capillary: 2.5 kV, sampling cone: 35 V, extraction cone: 4.0 V
Gas flow	Cone gas: 100 L/h, and desolvation gas: 800 L/h

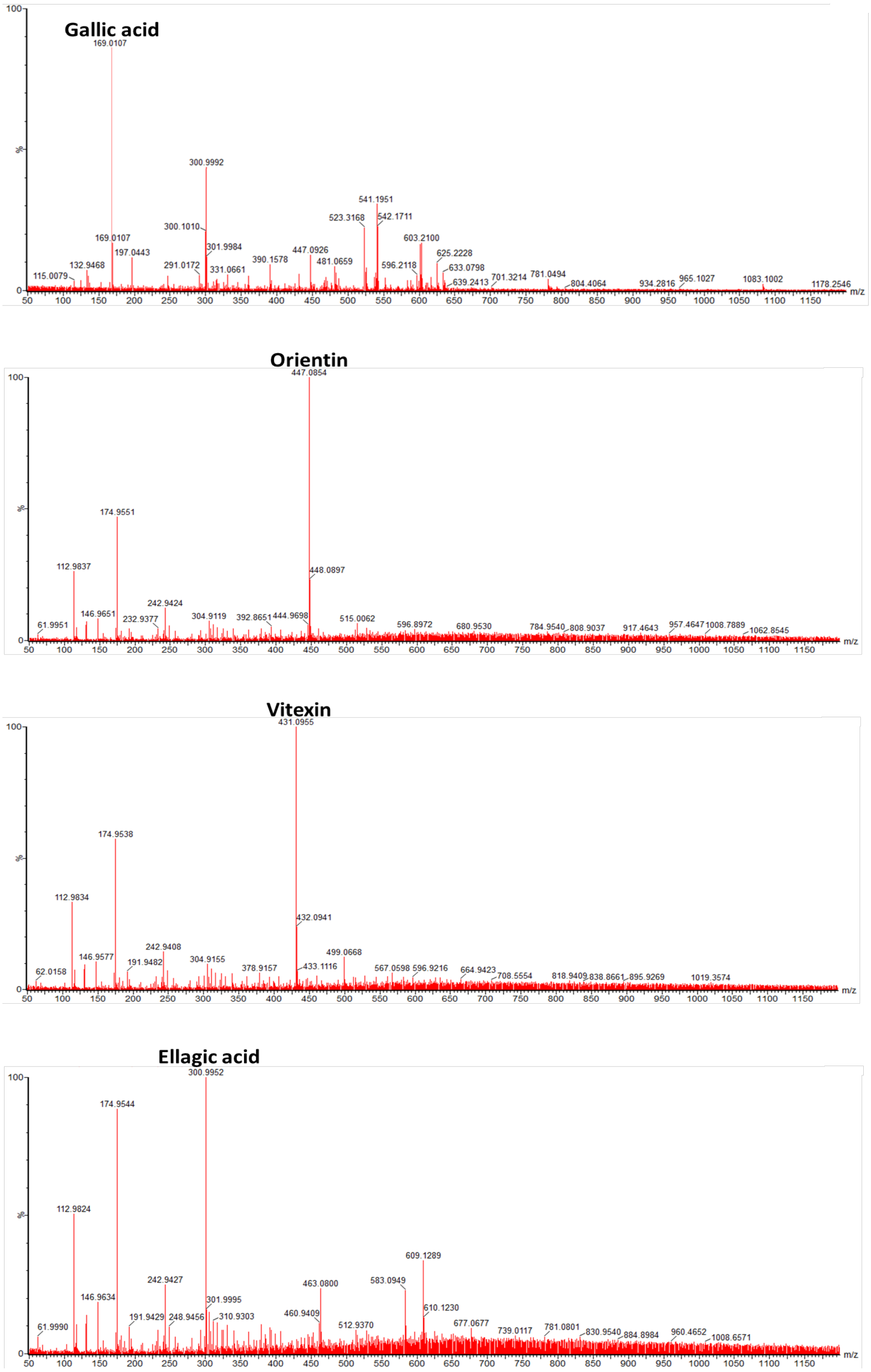

Figure S1. Mass chromatograms of major four phenolic compounds in TCE.
